# Apparent Ca Test Positivity and Crossmatch Reactivity in Donkeys from Italy: Intraspecies and Horse–Donkey Compatibility

**DOI:** 10.3390/ani16203204

**Published:** 2026-10-12

**Authors:** Chiara Bozzola, Enrica Zucca, Eva Spada, Luciana Baggiani, Roberta Perego, Daniela Proverbio

**Affiliations:** Dipartimento di Medicina Veterinaria e Scienze Animali (DIVAS), Università degli Studi di Milano, 26900 Lodi, Italy; chiara.bozzola@unimi.it (C.B.); enrica.zucca@unimi.it (E.Z.); luciana.baggiani@unimi.it (L.B.); roberta.perego@unimi.it (R.P.); daniela.proverbio@unimi.it (D.P.)

**Keywords:** donkey, *Equus asinus*, Ca blood type, blood group, crossmatch, transfusion compatibility, horse, Italy

## Abstract

Blood group distribution and serological compatibility are poorly characterized in donkeys. We estimated the apparent Ca positive-test frequency in 84 domestic donkeys and assessed donkey–donkey and horse–donkey crossmatch reactivity. Ca typing was performed using a commercial equine immunochromatographic assay. For donkey–donkey crossmatching, one major and one minor crossmatch were analyzed for each of the 77 Ca-concordant and 34 Ca-discordant pairs. Fifty-six of eighty-four donkeys yielded a positive result with the equine Ca assay (66.7%; 95% CI, 56.1–75.8%). Major combined macroscopic/microscopic reactivity occurred in 18/77 Ca-concordant and 11/34 Ca-discordant pairs, whereas minor combined reactivity occurred in 21/77 and 8/34 pairs, respectively. In crossed random-effects logistic models, the adjusted odds ratios for Ca-discordant versus Ca-concordant pairs were 1.56 (95% CI, 0.64–3.82) for major combined reactivity, 0.82 (95% CI, 0.32–2.10) for minor combined reactivity, and 1.57 (95% CI, 0.41–5.94) for major macroscopic reactivity; the minor macroscopic model was not estimable. Horse–donkey reactions were frequent and direction-dependent. When donkey plasma was tested against horse RBCs, combined reactivity occurred in 80/80 tests in the horse-donor/donkey-recipient dataset and in 76/76 tests in the reciprocal dataset, with macroscopic reactivity in 77/80 (96.3%) and 74/76 (97.4%), respectively. In the reciprocal horse-plasma/donkey-RBC direction, combined reactivity occurred in 53/80 (66.3%) and 51/76 (67.1%) tests, with macroscopic reactivity in 32/80 (40.0%) and 32/76 (42.1%), respectively. Ca concordance was not significantly associated with crossmatch reactivity in the present dataset, and Ca status alone did not account for the observed crossmatch-reactivity pattern.

## Simple Summary

Blood transfusion can be lifesaving in donkeys, but blood from a donor with a compatible blood type may still react with a recipient’s blood. We investigated Ca, the conventional designation of an equine red blood cell group factor, in 84 domestic donkeys from 21 farms or animal facilities in Italy and then tested blood compatibility among donkeys and between donkeys and horses. About two thirds of the domestic donkeys yielded a positive result with the equine Ca assay. Donkeys with the same Ca result could still show crossmatch reactions, indicating that this single blood group marker does not identify compatible donor–recipient pairs. Horse–donkey crossmatching frequently showed strong reactions. These findings support the use of blood typing to characterize potential donors, but crossmatching remains important before transfusion because it directly assesses whether donor and recipient blood react with each other.

## 1. Introduction

Blood transfusion is an important and potentially lifesaving therapeutic procedure in equine emergency and critical care, particularly in patients affected by acute or chronic hemorrhage, severe anemia, hemolysis, coagulopathies, or other conditions associated with inadequate oxygen-carrying capacity. However, transfusion of red blood cell (RBC)-containing products carries risks of immunological incompatibility, transfusion reactions, and reduced survival of transfused erythrocytes; appropriate donor selection and pretransfusion compatibility testing are therefore essential [1,2,3].

Red blood cell blood groups are determined by inherited, species-specific antigens expressed on the erythrocyte membrane. In horses, the A, C, D, K, P, Q, and U systems encompass numerous erythrocyte factors and generate a large number of possible phenotypes. Classical genetic and population studies have also shown that blood group frequencies vary among breeds and populations [4,5].

Among equine RBC antigens, Aa, Qa, and Ca have received particular attention in transfusion medicine. Aa and Qa are highly immunogenic, while naturally occurring antibodies against Aa and Ca have been reported; rapid pretransfusion identification of Ca and Aa has therefore been investigated in horses [6]. In a population of horses from Italy evaluated with the same Ca immunochromatographic platform used in the present study, 79.1% were Ca-positive, and anti-Ca alloantibodies were identified in 30.4% of Ca-negative horses [7].

In equids, donor selection is complicated by the diversity of erythrocyte antigens and by the practical impossibility of routinely typing every potentially relevant blood group factor. For this reason, blood typing and crossmatching answer different clinical questions: typing describes the antigenic profile of the donor and recipient for selected antigens, whereas crossmatching provides a functional assessment of whether antibodies present in plasma react with the erythrocytes of the other animal. Therefore, blood typing and crossmatching provide complementary rather than interchangeable information. Typing identifies selected erythrocyte antigens, whereas the major crossmatch directly tests recipient plasma against donor RBCs and the minor crossmatch tests donor plasma against recipient RBCs. Because complete antigen matching is generally not achievable in horses, current equine transfusion practice emphasizes crossmatching whenever feasible, particularly in previously transfused or potentially sensitized animals [1,2,8,9].

In horses, different crossmatch techniques, including conventional tube methods and more rapid stall-side approaches, have been investigated to support timely donor selection in emergency settings [2,8,9].

Compared with horses, transfusion medicine in donkeys remains poorly characterized [1,10]. Information regarding donkey erythrocyte antigen distribution, naturally occurring alloantibodies, and donor–recipient compatibility is limited, and clinical practice is frequently extrapolated from horses.

A further issue is antigenic divergence among equid species. Donkeys and mules express a species-associated erythrocyte antigen historically termed the ‘donkey factor’, which is present on donkey and mule RBCs but absent from horse RBCs. Antibodies directed against this antigen have been implicated in neonatal isoerythrolysis in mule foals [11,12,13]. These differences support direct compatibility testing when heterologous transfusion between equid species is contemplated.

The primary aims of the present study were to estimate the apparent Ca positive-test frequency in domestic donkeys and to determine whether Ca concordance was associated with serological crossmatch reactivity. Because donkey-specific blood-typing methods are not routinely available, the Ca immunochromatographic assay developed and previously used in horses was selected for the present study.

We hypothesized that Ca status would not fully account for crossmatch reactivity among donkeys and that horse–donkey testing would reveal additional serological incompatibility.

## 2. Materials and Methods

### 2.1. Study Design and Domestic Donkey Population

The epidemiological study population consisted of 84 domestic donkeys sampled from 21 farms, private holdings, or animal facilities in northern and central Italy. Source locations included holdings in the provinces of Milan, Bergamo, Brescia, Piacenza, Novara, Lodi, Verbano-Cusio-Ossola, and Pisa. The population was a convenience sample assembled during veterinary assessment and blood sampling carried out for evaluation of the animals’ health status.

Among the 84 domestic donkeys, 55 (65.5%) were females, including 5 pregnant jennies, and 29 (34.5%) were males, including 8 geldings. Age was available for 83 animals and ranged from 0.8 to 24 years (median, 7.0 years; IQR, 3.5–12.0 years). Body weight was available for 81 animals and ranged from 76 to 420 kg (median, 240 kg; IQR, 175–310 kg). Height at the withers was available for 81 animals and ranged from 93 to 144 cm (median, 114 cm; IQR, 105–128 cm). Body condition score was available for 81 animals and ranged from 2 to 4/5 (median, 3/5; IQR, 3–4/5). Most animals were crossbred; nine were recorded as purebred Amiata donkeys. Age and morphometric variables were used for descriptive signalment only and were not treated as epidemiological predictors of Ca phenotype. The five pregnant jennies were retained because pregnancy was not an exclusion criterion; pregnancy status was recorded descriptively and was not evaluated as a predictor. Medical history was obtained from owners, referring veterinarians, or available clinical records. None of the donkeys had previously received a blood transfusion.

At the time of sampling, 77/84 domestic donkeys were recorded as being in good general health. Animals with minor or chronic abnormalities were not automatically excluded because the epidemiological objective was to characterize Ca distribution in the sampled population rather than to define a healthy reference interval.

### 2.2. Horses Used for Interspecies Crossmatching

Crossmatching experiments additionally involved 11 horses aged 4.5–26 years. The horses represented a mixed clinical population and were not selected exclusively as healthy blood donors. Four were recorded as clinically healthy, six had a recorded clinical condition, and clinical status was unavailable for one horse. None of the horses had previously received a blood transfusion. Recorded breeds/types included Standardbred/Trotter, Arabian, indigenous-type, Appaloosa, Selle Français, Argentine, pony, and Haflinger horses. Sources included the University Equine Clinical Service. Individual signalment is reported in Table 1.

### 2.3. Blood Sample Collection and Processing

Whole blood was collected into EDTA tubes during clinically indicated veterinary assessment. Residual EDTA whole blood was used for Ca typing, and the corresponding EDTA plasma obtained after centrifugation was used for crossmatching.

### 2.4. Ca Blood Typing

Blood typing was performed on fresh blood or on samples stored at 4–6 °C and tested within 48 h of collection. Ca blood type was determined using an immunochromatographic method, validated for horses, with a monoclonal antibody (Lab Test Ca, Alvedia, Limonest France) following the same procedure previously described in horses [7]. Briefly, three drops of diluent were placed into a well, 10 µL of EDTA-anticoagulated whole blood were added and mixed with the diluent for 15 s, and the tip of the immunochromatographic strip containing Ca and control monoclonal antibodies at different positions was placed into the well for 2 min, allowing the erythrocyte suspension to migrate along the strip. A test was considered valid when a red band appeared at the control site. Absence of a band at the Ca position was interpreted as Ca-negative, whereas the presence of a visible Ca band was interpreted as Ca-positive. Reaction intensity was recorded semiquantitatively according to the laboratory worksheet; for epidemiological analysis, results were dichotomized as Ca-positive or Ca-negative according to the equine assay interpretation. Because the assay has not been validated against a donkey-specific reference method, these labels do not establish that the detected erythrocyte target is analytically or biologically identical across horses and donkeys.

### 2.5. Crossmatch Procedure

Major and minor crossmatches were performed using the same tube-agglutination procedure described previously for horses [8]. Briefly, a 4% washed erythrocyte suspension was prepared for each animal. For the major crossmatch, two drops of recipient plasma and two drops of donor erythrocyte suspension were mixed in a test tube; for the minor crossmatch, two drops of donor plasma and two drops of recipient erythrocyte suspension were used. Tubes were incubated at 37 °C for 15 min and centrifuged at approximately 1000× *g* for 15 s, then gently resuspended before macroscopic and microscopic evaluation. Macroscopic agglutination was recorded as positive or negative. Microscopic agglutination was graded semiquantitatively on the ordinal scale recorded in the laboratory worksheet (0, 0.5, 1, 2, 3, 4, or 5), representing progressively increasing erythrocyte aggregation. Rouleaux formation was assessed separately and was not considered equivalent to true agglutination. Because the clinical relevance of very weak microscopic reactions in donkeys is unknown, results were summarized using both a combined definition (any macroscopic agglutination or microscopic grade > 0) and macroscopic reactivity alone. All crossmatch reactions were evaluated by a single observer who was aware of Ca status. Intraobserver and interobserver reproducibility were not formally assessed.

### 2.6. Crossmatch Data Structure

Donkey–donkey tests were divided a priori into Ca-concordant combinations, in which the donor and recipient shared the same Ca phenotype and Ca-discordant combinations, in which their Ca phenotypes differed. For each donkey–donkey pair, both donor–recipient orientations were tested. Reciprocal records represented the same two immunological reactions with the major and minor crossmatches interchanged and were therefore not treated as independent observations. Horse–donkey crossmatches were classified according to both Ca phenotype combination and donor–recipient orientation. Four Ca phenotype combinations were identified: horse Ca+/donkey Ca+, horse Ca+/donkey Ca−, horse Ca−/donkey Ca+, and horse Ca−/donkey Ca−. Each phenotype combination could be evaluated in two reciprocal donor–recipient orientations, with either the horse or the donkey acting as the donor. Thus, Ca-discordant horse–donkey crossmatches included four directional combinations: horse Ca+ donor/donkey Ca− recipient, donkey Ca− donor/horse Ca+ recipient, horse Ca− donor/donkey Ca+ recipient, and donkey Ca+ donor/horse Ca− recipient. Major and minor crossmatch reactions were recorded separately for each donor–recipient orientation. Major and minor designations were based exclusively on the donor–recipient direction of plasma and RBC testing. Ca concordance or discordance was used only to stratify pairs and did not determine whether a reaction was classified as major or minor.

For pair-level analysis, one donor–recipient orientation was retained for each donkey–donkey pair. In Ca-discordant pairs, the retained orientation was Ca− donor/Ca+ recipient; in Ca-concordant pairs, the first recorded donor–recipient orientation was retained. Donkey–donkey combinations were generated within same-date sampling batches. All possible unordered within-batch combinations were tested in 8 of the 10 batches; the remaining two batches included 10 of 15 and 9 of 15 possible combinations, respectively. The 111 retained pairs involved 50 unique donkeys. Individual donkeys appeared in a median of 4 pairs (range, 2–9), and all 50 donkeys contributed to more than one pair. The 77 Ca-concordant pairs comprised 58 Ca+/Ca+ and 19 Ca−/Ca− pairs; the 34 discordant pairs comprised 34 Ca− donor/Ca+ recipient pairs. Positive combined major/minor reactions involved plasma from 33 of the 50 donkeys. The most frequently reactive donkey accounted for 5 of 58 positive directional reactions (8.6%), and the three most frequently reactive donkeys accounted for 12 of 58 (20.7%); marked clustering in a small number of animals was therefore not observed. The primary horse-donor/donkey-recipient dataset comprised 80 unique pairs. Reciprocal donkey-donor/horse-recipient testing was available for 76 unique pairs because insufficient sample material precluded reciprocal testing in four horse Ca+/donkey Ca− pairs. The anonymized pair-level dataset, including unique donor and recipient study IDs for all 111 retained donkey–donkey combinations, is provided as Supplementary Dataset S1.

### 2.7. Statistical Analysis

Statistical analyses were performed separately for the epidemiological evaluation of Ca distribution and for crossmatching. Categorical variables were summarized as counts and percentages. Continuous signalment variables were summarized by median, interquartile range (IQR), and minimum–maximum range. Age was treated as a descriptive signalment variable only and was not evaluated as a predictor of Ca phenotype. Associations between Ca phenotype and sex and between Ca phenotype and breed category (purebred Amiata vs. other breeds/crossbreds) were evaluated using two-sided Fisher’s exact tests.

The apparent Ca positive-test frequency was calculated as the number of domestic donkeys yielding a positive result with the equine Ca assay divided by the total number tested. The corresponding 95% confidence interval (CI) was calculated using the Wilson score method.

For each binary donkey–donkey crossmatch endpoint, the association with Ca discordance was evaluated using a penalized logistic mixed-effects model with Ca discordance as a fixed effect and crossed random intercepts for donor and recipient identity. Ca-concordant pairs were the reference category. Adjusted odds ratios with Wald-type 95% confidence intervals were reported. Fisher’s exact tests were retained as unadjusted sensitivity analyses. Models were estimated by maximum a posteriori optimization using a Laplace approximation and weakly informative regularization priors; Wald-type intervals and approximate two-sided *p* values were derived from the local covariance approximation. Model convergence, separation, and random-effect variance estimates were examined. When an endpoint had insufficient events or complete separation, no adjusted estimate was reported. Horse–donkey crossmatch results were summarized descriptively according to Ca phenotype combination and donor–recipient orientation. Agglutination grades were treated as ordinal semiquantitative observations rather than continuous measurements. All tests were two-sided, and *p* < 0.05 was used as the conventional threshold for statistical significance. Analyses were performed using Python 3.12 and statsmodels version 0.15.0.

## 3. Results

### 3.1. Domestic Donkey Population and Ca Typing

Of the 84 domestic donkeys, 56 yielded a positive result and 28 yielded a negative result with the equine Ca assay, corresponding to an apparent Ca positive-test frequency of 66.7% (Wilson 95% CI, 56.1–75.8%). Both assay-defined result categories were therefore well represented in the sampled population. No association was detected between Ca phenotype and sex (*p* = 0.811) or between Ca phenotype and breed category (purebred Amiata vs. other breeds/crossbreds; *p* = 0.468). Descriptive characteristics are summarized in Table 2.

### 3.2. Donkey–Donkey Crossmatches

Seventy-seven Ca-concordant donkey pairs and thirty-four Ca-discordant pairs were evaluated. In crossed random-effects logistic models, Ca discordance was not significantly associated with major combined reactivity (adjusted OR, 1.56; 95% CI, 0.64–3.82; *p* = 0.328), minor combined reactivity (adjusted OR, 0.82; 95% CI, 0.32–2.10; *p* = 0.679), or major macroscopic reactivity (adjusted OR, 1.57; 95% CI, 0.41–5.94; *p* = 0.509). Donor and recipient random-effect standard deviations were close to zero for all three estimable models, indicating boundary or singular fits; adjusted estimates were therefore very similar to the unadjusted comparisons. The minor macroscopic model was not estimable because only one positive event occurred among Ca-concordant pairs and none occurred among Ca-discordant pairs. Fisher’s exact tests were retained as unadjusted sensitivity analyses and did not alter the interpretation (Table 3).

### 3.3. Horse–Donkey Crossmatches

The primary horse-donor/donkey-recipient dataset comprised 80 unique pairs. The major crossmatch showed combined reactivity in all 80 pairs, irrespective of Ca concordance or discordance, and macroscopic agglutination in 77/80 (96.3%). The minor crossmatch showed combined reactivity in 53/80 pairs (66.3%) and macroscopic reactivity in 32/80 (40.0%) (Table 4). Reciprocal donkey-donor/horse-recipient testing comprised 76 unique pairs. In this orientation, the major crossmatch was reactive in 51/76 (67.1%) by the combined definition and in 32/76 (42.1%) macroscopically, whereas the minor crossmatch was reactive in 76/76 (100%) and 74/76 (97.4%), respectively. Thus, irrespective of whether the reaction was recorded as a major or minor crossmatch, reactivity was consistently more frequent when donkey plasma was tested against horse RBCs than in the reciprocal horse-plasma/donkey-RBC direction. The excluded second observation yielded the same binary combined and macroscopic classifications as the retained first observation in both donor–recipient orientations.

### 3.4. Signalment of Horses Used in Crossmatching

Individual signalment of the horses used for interspecies crossmatching is summarized in Table 1.

## 4. Discussion

This study integrates population-level Ca typing in domestic donkeys with serological crossmatch testing and extends a prevalence-based description toward a clinically relevant compatibility question. Three findings merit emphasis. First, approximately two thirds of the domestic donkeys yielded a positive result with the equine Ca assay. Second, both major and minor crossmatch reactivity occurred in Ca-concordant and Ca-discordant donkey pairs, without statistically significant differences between groups. Third, horse–donkey reactions were frequent and direction-dependent.

The apparent Ca positive-test frequency of 66.7% observed in the 84-donkey domestic cohort was lower than the 79.1% reported in the previously published Italian horse population tested with the same immunochromatographic platform [7]. The distribution of assay-defined positive and negative results also suggests that both categories are sufficiently represented in the sampled donkey population to be relevant for donor selection.

In addition, the Alvedia assay was developed for horses and has not been analytically validated against a donkey-specific reference method. Accordingly, the present percentage should be interpreted as an apparent positive-test frequency, not as a definitive estimate of true Ca-antigen prevalence in the Italian donkey population.

The donkey–donkey crossmatch findings support the concept that typing for a single antigen cannot establish donor–recipient serological compatibility. Major combined reactivity was observed in 23.4% of Ca-concordant and 32.4% of Ca-discordant pairs, whereas minor combined reactivity occurred in 27.3% and 23.5% of pairs, respectively. Ca concordance was not significantly associated with crossmatch reactivity in the present dataset. Ca status alone did not account for the observed crossmatch-reactivity pattern.

Reactivity among Ca-concordant pairs may reflect erythrocyte antigens other than Ca, naturally occurring or acquired alloantibodies, or methodological phenomena unrelated to clinically relevant alloimmunization. The current study did not identify antibody specificity and therefore cannot attribute these reactions to particular erythrocyte antigens. The results therefore support the use of crossmatching in addition to Ca typing when selecting potential blood donors. The absence of a significant difference between Ca-concordant and Ca-discordant pairs should not be interpreted as evidence that Ca has no immunohematological relevance in donkeys. Rather, it indicates that the Ca phenotype alone did not explain the serological compatibility pattern observed in this dataset. This distinction is important because crossmatch reactivity reflects the combined effect of multiple erythrocyte antigens and circulating antibodies, whereas Ca typing interrogates only a single antigenic system. The present findings therefore support a broader view of compatibility testing in donkeys, in which blood group typing contributes to donor characterization but cannot replace direct assessment of donor–recipient serological interaction. For example, even a Ca-positive donkey donor and a Ca-positive donkey recipient may show a reactive major crossmatch if recipient plasma contains antibodies against a donor erythrocyte antigen other than Ca. Conversely, the crossmatch result, rather than Ca status alone, provides the direct pair-specific serological assessment. Ca typing describes one selected erythrocyte factor and may assist donor characterization, but it cannot detect antibodies directed against other erythrocyte antigens. Crossmatching directly evaluates the serological interaction of a specific donor–recipient pair, although the clinical significance of weak microscopic reactions remains undetermined in donkeys.

The marked difference between combined macroscopic/microscopic reactivity and overt macroscopic agglutination warrants cautious interpretation. The combined endpoint classified any microscopic grade greater than zero as reactive, including very small aggregates. The clinical significance of weak microscopic reactions in donkeys is currently unknown, and no threshold linking the present grading scale to post-transfusion RBC survival or adverse reactions has been validated in this species. Accordingly, microscopic reactivity should be interpreted as evidence of laboratory serological interaction rather than necessarily as clinically relevant incompatibility.

Horse–donkey crossmatching showed frequent interspecies reactivity, particularly when donkey plasma was tested against horse RBCs. This directional pattern demonstrates species-associated serological divergence but cannot be attributed directly to the donkey factor. Because the donkey factor is expressed on donkey and mule erythrocytes and is absent from horse erythrocytes, it would more readily provide a mechanistic basis for antibodies reacting with donkey RBCs than for the predominant reaction of donkey plasma against horse RBCs observed here. The present findings may instead reflect naturally occurring or acquired antibodies in donkey plasma directed against one or more horse erythrocyte antigens that were not identified in this study. Therefore, the responsible antigen–antibody interactions remain unknown. These in vitro findings cannot predict the severity of an in vivo transfusion reaction or post-transfusion RBC survival.

Within its current analytical limitations, Ca testing may help characterize and organize potential donkey donors and preserve antigen information for future donor-panel development. The present data do not support using Ca status alone to declare a pair compatible. Major crossmatching remains the more direct pretransfusion assessment of recipient plasma against donor RBCs. The present findings support performing crossmatching whenever feasible, especially in previously transfused or potentially sensitized animals and before any contemplated interspecies transfusion; they do not establish a validated clinical decision threshold for weak microscopic reactions.

### Limitations

The domestic donkey population was a convenience sample obtained from non-randomly selected source populations and was not designed to be representative of all donkeys in Italy.

The commercial Ca immunochromatographic assay used in this study was developed for horses and has not been analytically validated against a donkey-specific reference method, flow cytometry, or molecular characterization. Therefore, the reported percentage should be interpreted as an apparent Ca positive-test frequency rather than as a definitive estimate of true Ca-antigen prevalence in donkeys.

Crossmatch pair selection was not random and was limited by sample availability. Some individual animals contributed to more than one donor–recipient pairing. Crossed random-intercept models were therefore fitted for donor and recipient identity. The estimated random-effect variances were close to zero, and the fitted models were boundary or singular solutions, reflecting the limited number of animals and sparse positive outcomes. Adjusted estimates should consequently be interpreted as exploratory. The minor macroscopic endpoint could not be modelled because only one positive event occurred. Larger prospective studies with balanced selection are needed to confirm the relationship between Ca phenotype and serological compatibility.

The clinical significance of weak microscopic reactions has not been established in donkeys. Because the combined endpoint classified every microscopic grade >0 as reactive, it may overestimate clinically meaningful incompatibility. Future studies should prospectively compare tube and gel-based methods, assess interobserver repeatability, define donkey-specific analytical thresholds, and ideally relate crossmatch grades to post-transfusion RBC survival and clinical reactions. Crossmatch interpretation was performed by a single observer who was not blinded to Ca status. The semiquantitative microscopic grading system was not evaluated for intraobserver or interobserver reproducibility.

## 5. Conclusions

In this population of 84 domestic donkeys from Italy, approximately two thirds yielded a positive result with the equine Ca assay. Ca concordance was not significantly associated with crossmatch reactivity in the present dataset. Both major and minor crossmatch reactions were observed among Ca-concordant and Ca-discordant donkey pairs. The present data do not support using Ca status alone to declare a pair compatible. Horse–donkey crossmatches showed frequent interspecies reactivity, further supporting the need for caution when heterologous transfusion is considered.

Taken together, these findings support a two-level approach to pretransfusion testing in donkeys, involving Ca testing for donor characterization and direct major crossmatching for a specific donor–recipient pair. Where a suitable donkey donor is available, homologous donation is the more conservative option. The present in vitro study does not establish the clinical safety of horse blood for donkeys or define circumstances in which heterologous transfusion is acceptable. If emergency horse-to-donkey transfusion is contemplated, direct crossmatching is essential, but a non-reactive in vitro result would not by itself demonstrate clinical safety. Because no mules were tested, these results cannot be directly extrapolated to mule donor selection or transfusion compatibility; dedicated studies are required.

## Figures and Tables

**Table 1:** Signalment, Ca assay result, and Ca reaction grade of the 11 horses used for horse–donkey crossmatching.

Horse	Source	Breed/Type	Sex	Age (yr)	Ca	Ca Reaction Grade
Omer	University Equine Clinical Service	Standardbred/Trotter	M	10.0	Ca+	5
Zingara	University Equine Clinical Service	Standardbred/Trotter	F	12.0	Ca+	5
Queen	Agr. Roccolo–Angolo Terme (BS)	Arabian	F	12.0	Ca+	5
Luna	Agr. Roccolo–Angolo Terme (BS)	Arabian	F	17.0	Ca+	5
Lola	Agr. Roccolo–Angolo Terme (BS)	Indigenous type	F	9.0	Ca+	5
Fiordaliso	Agr. Roccolo–Angolo Terme (BS)	Appaloosa	F	13.0	Ca+	5
It’s Me	Maneggio Eden	Selle Français	M	26.0	Ca−	0
Magie	University Equine Clinical Service	Selle Français	F	22.0	Ca+	5
Brattas	University Equine Clinical Service	Argentine	F	15.0	Ca+	5
Sissi	University Equine Clinical Service	Pony	F	19.0	Ca−	0
Bionda	University Equine Clinical Service	Haflinger	F	4.5	Ca+	5

**Table 2:** Descriptive characteristics of the domestic donkey population.

Characteristic	Category	Result
Sex (n = 84)	Female, total	55 (65.5%)
	of which pregnant	5/55 (9.1%)
	Male, total	29 (34.5%)
	of which geldings	8/29 (27.6%)
Age, years (n = 83)	Median (IQR); range	7.0 (3.5–12.0); 0.8–24
Body weight, kg (n = 81)	Median (IQR); range	240 (175–310); 76–420
Height at withers, cm (n = 81)	Median (IQR); range	114 (105–128); 93–144
BCS/5 (n = 81)	Median (IQR); range	3 (3–4); 2–4
Breed (n = 82)	Purebred Amiata	9 (11.0%)
	Other breeds/crossbreds	73 (89.0%)
Health status (n = 84)	Good general health	77 (91.7%)
	Minor/chronic abnormalities	7 (8.3%)

IQR, interquartile range; BCS, body condition score. Age was unavailable for one donkey; body weight, height at the withers, and BCS were unavailable for three donkeys; and breed information was unavailable for two donkeys. Missing age and morphometric data were not available in the source records and could not be recovered from the owners. Breed was recorded as missing when it was unknown to the owner and could not be reliably identified. Percentages were calculated using the available denominator shown for each variable.

**Table 3:** Major and minor crossmatch reactivity in Ca-concordant and Ca-discordant donkey pairs.

Crossmatch Endpoint	Ca-Concordant (n = 77), Positive n (%)	Ca-Discordant (n = 34), Positive n (%)	Fisher *p*	Adjusted OR (95% CI), *p*
Major—combined macro/micro	18 (23.4)	11 (32.4)	0.354	1.56 (0.64–3.82), *p* = 0.328
Minor—combined macro/micro	21 (27.3)	8 (23.5)	0.816	0.82 (0.32–2.10), *p* = 0.679
Major—macroscopic only	6 (7.8)	4 (11.8)	0.492	1.57 (0.41–5.94), *p* = 0.509
Minor—macroscopic only	1 (1.3)	0 (0.0)	1.000	Not estimable

Note: Ca-concordant pairs included Ca+/Ca+ and Ca−/Ca− combinations; Ca-discordant pairs consisted of Ca+/Ca− combinations. Combined reactivity was defined as macroscopic agglutination and/or microscopic grade > 0. Adjusted ORs compare Ca-discordant with Ca-concordant pairs and were obtained from logistic mixed-effects models with crossed random intercepts for donor and recipient identity. The minor macroscopic model was not estimable because of complete separation and insufficient positive events.

**Table 4:** Horse–donkey crossmatch reactivity according to donor–recipient orientation and Ca phenotype combination.

Direction	Horse Ca	Donkey Ca	n	Major Combined n (%)	Major Macro n (%)	Minor Combined n (%)	Minor Macro n (%)
Horse → donkey	Ca+	Ca+	42	42/42 (100.0)	42/42 (100.0)	22/42 (52.4)	9/42 (21.4)
	Ca+	Ca−	23	23/23 (100.0)	21/23 (91.3)	18/23 (78.3)	13/23 (56.5)
	Ca−	Ca+	6	6/6 (100.0)	5/6 (83.3)	4/6 (66.7)	2/6 (33.3)
	Ca−	Ca−	9	9/9 (100.0)	9/9 (100.0)	9/9 (100.0)	8/9 (88.9)
**Total**	**—**	**—**	**80**	**80/80** (**100.0**)	**77/80** (**96.3**)	**53/80** (**66.3**)	**32/80** (**40.0**)
Donkey → horse	Ca+	Ca+	42	22/42 (52.4)	9/42 (21.4)	42/42 (100.0)	42/42 (100.0)
	Ca+	Ca−	19	16/19 (84.2)	13/19 (68.4)	19/19 (100.0)	18/19 (94.7)
	Ca−	Ca+	6	4/6 (66.7)	2/6 (33.3)	6/6 (100.0)	5/6 (83.3)
	Ca−	Ca−	9	9/9 (100.0)	8/9 (88.9)	9/9 (100.0)	9/9 (100.0)
**Total**	**—**	**—**	**76**	**51/76** (**67.1**)	**32/76** (**42.1**)	**76/76** (**100.0**)	**74/76** (**97.4**)

**Table footnote: Major crossmatch** represents recipient plasma against donor RBCs, whereas **minor crossmatch** represents donor plasma against recipient RBCs. Accordingly, in the horse-donor/donkey-recipient orientation, the major crossmatch corresponds to donkey plasma against horse RBCs and the minor crossmatch to horse plasma against donkey RBCs. In the reciprocal donkey-donor/horse-recipient orientation, these immunological directions are reversed. **Combined reactivity** was defined as macroscopic agglutination and/or microscopic agglutination grade > 0; macroscopic reactivity is reported separately.

## Data Availability

The anonymized pair-level donkey–donkey dataset used for the crossed random-effects analyses is provided as Supplementary Dataset S1. Additional anonymized data supporting the study are available from the corresponding author upon reasonable request, subject to institutional and owner-consent requirements.

## Supplementary Materials

The following supporting information can be downloaded at: https://www.mdpi.com/article/10.3390/ani16203204/s1, Supplementary Dataset S1. Anonymized donkey–donkey pair-level dataset and crossed random-effects logistic-model results.

## Abbreviations

The following abbreviations are used in this manuscript:

Abbreviation	Definition
BCS	body condition score
CI	confidence interval
EDTA	ethylenediaminetetraacetic acid
IQR	interquartile range
RBC	red blood cell
